# MicroRNA Regulation of the Estrogen Receptor Alpha 3′UTR in Canine Mammary Tumor (CMT28) Cells

**DOI:** 10.3390/vetsci13101071

**Published:** 2026-10-10

**Authors:** Deepika Goyal, Richard Curtis Bird

**Affiliations:** Department of Pathobiology, College of Veterinary Medicine, Auburn University, Auburn, AL 36849, USA; dzg0048@auburn.edu

**Keywords:** canine mammary tumor, miR-18a, miR-19a, estrogen receptor 1 (*ESR1*), luciferase reporter assay, 3′ untranslated region, post-transcriptional regulation

## Abstract

Reduced estrogen receptor alpha (ERα/*ESR1*) expression is seen in canine mammary tumors, but the mechanisms involved are not fully understood. This study examined *ESR1*, miR-18a, and miR-19a expression in canine mammary cell lines. Further CMT28, a canine mammary tumor cell line, was used to test whether these miRNAs can regulate the reporter activity through selected regions of the canine *ESR1* 3′UTR containing predicted miR-18a and miR-19a binding sites. *ESR1* mRNA expression was measured by RT-qPCR, and miR-18a and miR-19a expression was measured using LNA-based assays. *ESR1* mRNA expression was much lower in CMT27, CMT28, and CMT47 compared with normal canine mammary epithelial cells, while CMT12 showed higher expression. miR-18a and miR-19a were increased to different levels across the tumor cell lines. Bioinformatic analysis identified predicted miR-18a and miR-19a seed-match sites in the canine *ESR1* 3′UTR. Luciferase reporter assays showed that both miR-18a and miR-19a significantly reduced activity of the reporter containing selected regions of the 3′UTR of *ESR1* encoding predicted miR-18a and miR-19a binding sites. In contrast, neither miRNA significantly reduced activity of the mutant reporter containing deletions in the predicted miR-18a and miR-19a seed-match regions. Anti-miR-18a and anti-miR-19a also significantly increased reporter activity compared with the corresponding miRNA alone. These findings provide functional evidence that miR-18a and miR-19a can regulate reporter activity through selected regions of the canine *ESR1* 3′UTR. Further studies are needed to determine the role of this regulation in endogenous *ESR1* expression and canine mammary tumor biology.

## Simple Summary

Mammary tumors are common in female dogs, and estrogen can play an important role in their growth and behavior. Some tumors have reduced estrogen receptor expression, but the reasons for this are not fully understood. In this study, we examined whether two small regulatory molecules, miR-18a and miR-19a, could affect reporter activity through selected regions of the canine *ESR1* 3′UTR. MicroRNAs can bind to messenger RNA and reduce gene activity. We found that both miR-18a and miR-19a reduced activity of a reporter containing selected regions of the canine *ESR1* 3′UTR. This effect was not seen when the predicted miRNA target regions were deleted. Blocking the miRNAs also increased reporter activity. These findings suggest that miR-18a and miR-19a can affect reporter activity through selected regions of canine *ESR1* 3′UTRs and provide a basis for further studies in canine mammary cancer.

## 1. Introduction

Breast cancer is one of the most frequently diagnosed cancers in women, and mammary tumors are also common in intact female dogs [1,2,3]. Both species share several important biological and clinical features, including spontaneous tumor development, marked heterogeneity, and hormone-dependent growth [4]. Because of these similarities, canine mammary tumors (CMTs) are often used as a spontaneous comparative model to understand mammary tumorigenesis and hormone-driven cancer progression in women [2,5].

There are two types of estrogen receptors—estrogen receptor alpha (ERα) and estrogen receptor beta (ERβ), encoded by the *ESR1* and *ESR2* genes, respectively. ERα and ERβ have some similar functions, but they can also have different effects depending on the tissue and cell type. In some tissues, ERβ can oppose ERα mediated signaling [6]. Both ERα and ERβ have been detected in canine mammary tissues and tumors, although their expression varies among normal, benign, and malignant tissues [7,8]. This study focuses on ERα, encoded by *ESR1*. When estradiol binds to ERα, it forms a dimer and moves into the nucleus, where it regulates genes involved in cell growth, differentiation, and survival. Based on ERα, PR, and HER2 expression profiles, CMTs have been grouped into phenotypes resembling luminal A, luminal B, HER2-positive, and triple-negative breast cancer [3,9]. However, these canine groups may not fully correspond to the established human breast cancer subtypes. In human breast cancer, tumors that express ERα usually respond better to endocrine therapies, including aromatase inhibitors and selective estrogen receptor modulators such as letrozole and tamoxifen [10,11]. Tumors with reduced or abnormal ERα expression tend to be more aggressive and are associated with poorer outcomes in both species [10,12].

In human breast cancer, ERα loss can occur through several mechanisms, including promoter methylation, *ESR1* mutations, and post-transcriptional regulation. However, the mechanisms underlying ERα downregulation in CMTs are not well understood, and the post-transcriptional regulation of *ESR1* in dogs remains unclear.

MicroRNAs (miRNAs) are small, highly conserved non-coding RNAs, about 18–25 nucleotides long, that regulate gene expression after transcription by binding to complementary sequences in the 3′UTR of target mRNAs [13,14]. Abnormal miRNA expression is common in many cancers, including CMTs, and can influence cell growth, apoptosis, invasion, and metastasis [5,14,15,16]. Among these, miR-18a and miR-19a—both part of the miR-17–92 cluster—have been linked to mammary tumor progression [17,18]. In both human breast cancer and CMTs, higher miR-18a expression is associated with reduced *ESR1*/ERα expression [19,20,21]. miR-19a has also been reported to be elevated in early breast cancer and CMTs [15,22,23]. Several studies in human breast cancer suggest that miRNA-mediated suppression of *ESR1* may contribute to resistance to endocrine therapy [21,24,25]. Since miRNA expression patterns in CMTs share similarities with those reported in human breast cancer, miR-18a and miR-19a may similarly influence estrogen receptor signaling in dogs [5,26].

We selected miR-18a and miR-19a based on findings from previous studies by our research group. Both miRNAs were identified by cell-line and exosomal miRNA profiling and were found at higher levels in CMT cells and their secreted exosomes compared with normal canine mammary epithelial cells (CMECs) [15,22]. In a later clinical study of dogs with mammary carcinoma, circulating miR-18a was identified as a potential prognostic biomarker, with higher levels associated with lymphatic invasion [18]. Bioinformatic analyses also identified the canine *ESR1* transcript as a predicted target of both miRNAs [22,27]. However, expression patterns and target predictions do not establish a functional regulatory interaction. To our knowledge, the effects of these miRNAs on a reporter containing canine *ESR1* 3′UTR regions have not previously been reported.

In this study, we examined whether miR-18a and miR-19a can affect reporter activity through selected regions of the canine *ESR1* 3′UTR in mammary tumor cells. We measured endogenous expression of *ESR1*, miR-18a, and miR-19a. Luciferase reporter assays containing the selected wild-type *ESR1* 3′UTR regions and a corresponding mutant *ESR1* 3′UTR reporter having deletions within the predicted miR-18a and miR-19a seed-match regions were used to assess miRNA-dependent reporter activity. We also used miRNA expression vectors and anti-miRs to assess specificity of these effects.

## 2. Materials and Methods

### 2.1. Cell Culture

Canine mammary tumor (CMT) cell lines CMT12, CMT27, CMT28, and CMT47 were used in this study. These cell lines were selected because they are commonly used CMT models and have been used in previous studies of miRNA expression [15,22]. These cell lines were originally derived from mammary tumors from individual dogs, as described previously [28,29]. CMT12 was derived from adenocarcinoma in a poodle, CMT27 from a carcinoma in a German Shepherd, CMT28 from an adenocarcinoma in a mixed-breed dog, and CMT47 from an adenocarcinoma in a Miniature Schnauzer [22,28]. Canine mammary epithelial cells (CMECs), used as normal mammary epithelial reference cells, were derived from mammary glands of one female Beagle at necropsy with no prior history of mammary carcinoma [29,30]. The CMT cell lines were used between passages 20 and 50. The CMECs represented a single primary cell culture and were used below passage 15. We identified each cell line using the labels and records of the original frozen stocks. Cell morphology was regularly checked during culture, and each cell line maintained its distinct appearance. No additional molecular authentication was performed in this study. Cells were grown in Dulbecco’s Modified Eagle Medium (DMEM) supplemented with L-glutamine (Quality Biological, Gaithersburg, MD, USA), 10% fetal bovine serum (Atlas Biologicals, Fort Collins, CO, USA), and 1% penicillin-streptomycin (Sigma-Aldrich, St. Louis, MO, USA), and were maintained at 37 °C in a humidified incubator with 5% CO_2_. Cells were routinely passaged after reaching approximately 70–80% confluence. All cells were handled in standard vented-lid 75 cm^2^ tissue-culture flasks (VWR Avantor, Radnor, PA, USA). The cell cultures were examined for Mycoplasma contamination by electron microscopy at the time of cryopreservation. Routine Mycoplasma testing was not performed during the present study. Work involving canine cell lines was performed under protocols approved by the Auburn University Institutional Animal Care and Use Committee.

### 2.2. RNA Extraction

Total RNA was extracted using the miRNeasy kit with QIAzol lysis reagent and RNase-free DNase (Qiagen, Germantown, MD, USA) according to the manufacturer’s instructions. Cells were collected at 70–80% confluence and lysed directly in QIAzol. RNA was purified using miRNeasy spin columns, eluted in RNase-free water, and stored at −80 °C until further use. RNA concentration and purity were assessed using a NanoDrop spectrophotometer (Thermo Fisher Scientific, Waltham, MA, USA), and only samples with A260/A280 ratios between 1.8 and 2.0 were used for cDNA synthesis and qPCR.

### 2.3. cDNA Synthesis

For mRNA targets, including *ESR1* and the reference gene *GAPDH*, cDNA was synthesized using the High-Capacity RNA-to-cDNA Kit (Thermo Fisher Scientific, Waltham, MA, USA). Each reaction contained 1 µg of total RNA and was incubated at 37 °C for 60 min, followed by heat inactivation at 95 °C for 5 min. The resulting cDNA was stored undiluted at −20 °C and diluted twofold immediately before qPCR.

For miRNA analysis, cDNA was synthesized using the Qiagen miRCURY LNA miRNA RT Kit. Each reaction contained approximately 200 ng of total RNA and was incubated at 42 °C for 60 min, followed by heat inactivation at 95 °C for 5 min. The resulting undiluted cDNA was stored at −20 °C and diluted 60-fold immediately before qPCR.

### 2.4. RT-qPCR

Using the diluted cDNA as described above, RT-qPCR reactions for mRNA and miRNA targets were performed separately on a Bio-Rad CFX96 Real-Time PCR Detection System (Bio-Rad Laboratories, Hercules, CA, USA). Each reaction was run in duplicate. No-reverse-transcription (no-RT) and no-template controls were included in all runs to assess genomic DNA and reagent contamination.

To assess mRNA expression of *ESR1*, and *GAPDH* reactions were prepared using the PowerUp SYBR Green Master Mix (Thermo Fisher Scientific, Waltham, MA, USA) with gene-specific primers (Table 1). The *GAPDH* primer sequences were based on a previously published study [9,31], while the *ESR1* primers were designed using NCBI Primer-BLAST to ensure the amplicon spanned an exon–exon junction. All primers were synthesized by IDT (Integrated DNA Technologies, Coralville, IA, USA). The *ESR1* amplicon was sequence-verified by conventional PCR followed by Sanger sequencing (MGH DNA Core, Massachusetts General Hospital, Boston, MA, USA) to confirm target specificity. Annealing conditions for *ESR1* were optimized using a serial dilution curve, and the final reactions were run at 60.5 °C, yielding clean, specific amplification and consistent Ct values. The reaction was run under the following cycling conditions: 50 °C for 2 min, 95 °C for 2 min, followed by 36 cycles of 95 °C for 1 s and 60.5 °C for 30 s. A melt-curve step was included to confirm amplicon specificity. *GAPDH* was used as the endogenous reference gene to normalize the *ESR1* mRNA expression. We selected *GAPDH* because it has been used in previous studies of canine mammary cancer cell lines [31]. However, we did not formally compare its stability with other reference genes using geNorm or NormFinder.

For miRNA expression, RT-qPCR was performed using the miRCURY LNA miRNA PCR Kit (Qiagen, Germantown, MD, USA) with LNA primer assays for cfa-miR-18a-5p (Assay ID: YP00204207), cfa-miR-19a-3p (Assay ID: YP00205862), and U6 snRNA (Assay ID: YP02119464). Cycling conditions were 95 °C for 2 min, followed by 40 cycles of 95 °C for 10 s and 56 °C for 1 min. U6 snRNA was used as the endogenous reference for miRNA normalization, as reported in a previous CMT study [32]. ΔCt values were calculated relative to U6 Ct. Relative expression was calculated using the ΔCt method, and fold-change values were derived using the 2^−ΔΔCt^ method.

Amplification efficiencies were calculated from the slopes of standard curves generated using serially diluted cDNA. The amplification efficiencies were 104.6% for *GAPDH*, 105.3% for *ESR1*, 105.1% for miR-18a, 90.4% for miR-19a, and 91.9% for U6. The corresponding standard curves with R^2^ values are provided in Supplementary Figures S1 and S2.

### 2.5. In Silico Identification of miRNA Binding Sites

Before designing the luciferase constructs, the canine *ESR1* 3′UTR was examined for predicted miRNA binding sites using the miRDB database (http://mirdb.org; initially accessed on 24 June 2023 and rechecked on 14 September 2023). miRDB prediction scores ranged from 50 to 100, and scores above 80 were generally considered high-confidence targets [27]. Both cfa-miR-18a and cfa-miR-19a appeared among the top predicted regulators, with prediction scores of 99 and 96, respectively. TargetScanHuman 8.0 (TargetScanHuman 8.0; initially accessed on 24 June 2023 and rechecked on 14 September 2023) was also used to examine the location and conservation of predicted miR-18a and miR-19a seed-match sites within the canine *ESR1* 3′UTR (NM_001286958.2) [33,34]. Dog was selected as the species, and miR-18 and miR-19 were searched separately. Site locations and conservation categories were taken directly from TargetScan and reported as “Conserved” or “Poorly conserved.”

Based on these results, the *ESR1* 3′UTR segments containing the predicted binding sites were selected for cloning into the luciferase reporter vector to experimentally assess their regulatory activity.

### 2.6. Plasmids and miRNA Constructs

The *ESR1* 3′UTR reporter constructs, and precursor miRNA expression vectors used in this study were obtained from GeneCopoeia (Rockville, MD, USA). The reporter construct containing selected wild-type *ESR1* 3′UTR regions (CS-miT740T-MT05-01) contained an 859 bp insert derived from the canine *ESR1* 3′UTR (NM_001286958.2) cloned downstream of the Gaussia luciferase reporter gene in the pEZX-MT05 vector. The insert contained three selected non-overlapping *ESR1* 3′UTR regions containing predicted miR-18a and miR-19a seed-match sites. Fragment 1 was 308 bp and contained the predicted miR-18a binding region, Fragment 2 was 307 bp and contained the predicted miR-19a binding region, and Fragment 3 was 244 bp and contained overlapping predicted binding regions for both miRNAs. Hereafter, this construct is referred to as the *ESR1*-WT reporter. “*ESR1*-WT” refers to the unmodified sequences of the selected regions and does not indicate the complete native *ESR1* 3′UTR.

A corresponding mutant *ESR1* 3′UTR reporter construct (CS-miT740T-MT05-02) was used as a negative control. Relative to *ESR1*-WT reporter, this construct contained three deletions within the selected *ESR1* 3′UTR regions: GCACCTTA at nucleotides 151–158, TTTGCAC at nucleotides 459–465, and GCACCTT at nucleotides 766–772 of the reporter insert. These deletions removed the three predicted miRNA seed-match sites and reduced the insert length from 859 to 837 bp. Hereafter, this construct is referred to as the *ESR1*-MUT reporter.

CMT28 cells were transfected with precursor miRNA vectors for cfa-miR-18a (CS-miR738T-MR04-01) and cfa-miR-19a (CS-miR739T-MR04-01) for the reporter assays. The miR-18a vector contained an eGFP reporter, whereas miR-19a vector contained an mCherry reporter. A GeneCopoeia miRNA control vector (miR-C; CmiR0001-MR04) containing eGFP was used as the negative control for miRNA vector transfection. The precursor miRNA and fluorescent reporter protein were expressed from the same vector under the same promoter. Therefore, GFP and mCherry fluorescence provided indirect evidence of expression of the corresponding miRNA vectors but did not directly confirm increased levels of mature miR-18a or miR-19a. For rescue experiments, miArrest™ synthetic miRNA inhibitors, containing antisense sequences, (GeneCopoeia) targeting miR-18a-5p (HmiR-AN0255-SN-5) and miR-19a-3p (HmiR-AN0293-SN-5) were used. Both inhibitors were single-stranded, 2′-O-methyl-modified RNA oligonucleotides. Although these inhibitors were designed for human miR-18a-5p and miR-19a-3p, the mature sequences of both miRNAs are identical in humans and dogs. Therefore, the inhibitors are also complementary to cfa-miR-18a-5p (MIMAT0009832) and cfa-miR-19a-3p (MIMAT0006650). A non-targeting anti-miR control was not included in the rescue experiments. Additional information on the reporter constructs, miRNA expression vectors, inhibitors and *ESR1* 3′UTR sequences is provided in Supplementary Tables S1–S3.

### 2.7. Plasmid Preparation

Mix & Go! DH5α competent *E. coli* (Zymo Research, Irvine, CA, USA) was transformed with the *ESR1* 3′UTR reporter constructs and miRNA expression plasmids according to the manufacturer’s instructions. Transformed colonies were grown overnight in 2YT broth containing ampicillin at 37 °C with shaking. Plasmid DNA was purified using the ZymoPURE Midiprep Kit (Zymo Research, Irvine, CA, USA) according to the manufacturer’s instructions. Plasmid concentrations typically ranged from 1.5 to 3 µg/µL, with A260/A280 ratios between 1.8 and 2.0.

### 2.8. Transient Transfection and Luciferase Reporter Assay

CMT28 cells were seeded in 24-well plates at ~1.5 × 10^5^ cells/mL (500 µL/well) and allowed to reach 60–80% confluence. CMT28 cells were selected for the functional reporter assays because they showed relatively low endogenous miR-18a and miR-19a expression and were well suited for reproducible transfection based on their growth and culture characteristics. For rescue experiments, cells were first transfected with 5 pmol of anti-miR oligonucleotides using Lipofectamine RNAiMAX (Thermo Fisher Scientific, Waltham, MA, USA). After approximately 18 h, the transfection medium was replaced with fresh complete medium.

Twenty-four hours after the initial anti-miR transfection, cells were transfected with either 0.5 µg of reporter plasmid alone or co-transfected with 0.25 µg of reporter plasmid and 0.25 µg of the corresponding miRNA expression vector or miR-C control vector using Lipofectamine 3000 (Thermo Fisher Scientific, Waltham, MA, USA) in Opti-MEM. The total amount of plasmid DNA was therefore maintained at 0.5 µg per well.

Forty-eight hours after plasmid transfection, conditioned media were collected, and Gaussia luciferase activity was measured using the GeneCopoeia Dual Luminescence Assay Kit. Luminescence was recorded using a Synergy HTX multimode microplate reader (BioTek, Winooski, VT, USA; Gen5 software (version 2.09.1)). Gaussia luciferase activity was normalized to secreted alkaline phosphatase (SEAP) activity to account for transfection efficiency. All transfection and luciferase reporter experiments were performed in three independent experimental runs, with two transfected wells per condition in each run. Luminescence from each well was measured in duplicate.

### 2.9. Fluorescence Imaging

An EVOS fluorescence imaging system (Thermo Fisher Scientific, Waltham, MA, USA) was used to confirm expression of the fluorescent reporter proteins following transfection of the miRNA expression vectors in CMT28 cells. GFP and RFP filter cubes were used to detect eGFP and mCherry fluorescence, respectively, with a 10× objective lens. GFP images were taken at 60% illumination with an exposure time of 250 ms, while RFP images were taken at 60% illumination with an exposure time of 750 ms. The images were used only to confirm fluorescent reporter expression.

### 2.10. Statistical Analysis

RT-qPCR experiments were performed in four independent experimental runs, with two technical replicates per condition in each run. Technical replicates were averaged within each run before statistical analysis. Statistical analyses were performed in R (version 4.3).

For qPCR experiments, *ESR1* expression was normalized to *GAPDH*, whereas miR-18a and miR-19a expression was normalized to U6. ΔCt values were calculated as the difference between the Ct value of the target and the corresponding reference gene. ΔCt values were analyzed using linear mixed-effects models with the lmerTest package, with cell line included as a fixed effect and experimental run included as a random intercept. Estimated marginal means (EMMs) were obtained using the emmeans package, and each CMT cell line was compared with CMEC using Dunnett-adjusted contrasts. Relative fold changes were calculated from the model-estimated differences using the 2^−ΔΔCt^ method, with CMEC used as the reference. Normality of the model residuals was assessed using residual Q-Q plots and the Shapiro–Wilk test, and equality of variances was assessed using Levene’s test.

Luciferase reporter experiments were performed in three independent runs, with two transfected wells per condition in each run. Each independent experimental run was performed on a different day using a new cell culture plate and a separate transfection. Each well was measured in duplicate. Incomplete GLuc/SEAP read pairs were excluded, and valid technical measurements were averaged within each run before statistical analysis. The independent experimental run was treated as the statistical unit (*n* = 3). Gaussia luciferase activity was normalized to SEAP activity, and the reporter-only condition was set to 1 as the reference. Because reporter-only activity was used for normalization, it was not included in the statistical comparisons. The effects of reporter construct (ESR1-WT or ESR1-MUT), miRNA treatment (miR-C, miR-18a, or miR-19a), and their interaction were analyzed using a two-way repeated-measures ANOVA with experimental run treated as the repeated measure. Comparisons between each miRNA treatment and miR-C were performed using two-sided paired *t*-tests. The effects of miR-18a and miR-19a were also compared between the ESR1-WT and ESR1-MUT using paired *t*-tests. Rescue experiments were analyzed using two-sided paired *t*-tests comparing each miRNA treatment with the corresponding miRNA plus anti-miR treatment. Holm adjustment was used for the two ESR1-WT-versus-ESR1-MUT comparisons. Effect estimates, 95% CIs, and exact *p* values were reported. Model residuals were assessed using a Q-Q plot and the Shapiro–Wilk test. A *p* value < 0.05 was considered statistically significant. Run-level reporter data and effect estimates with 95% CIs are provided in Supplementary Table S4.

## 3. Results

### 3.1. Canine Mammary Cell Lines

**Figure 1:**
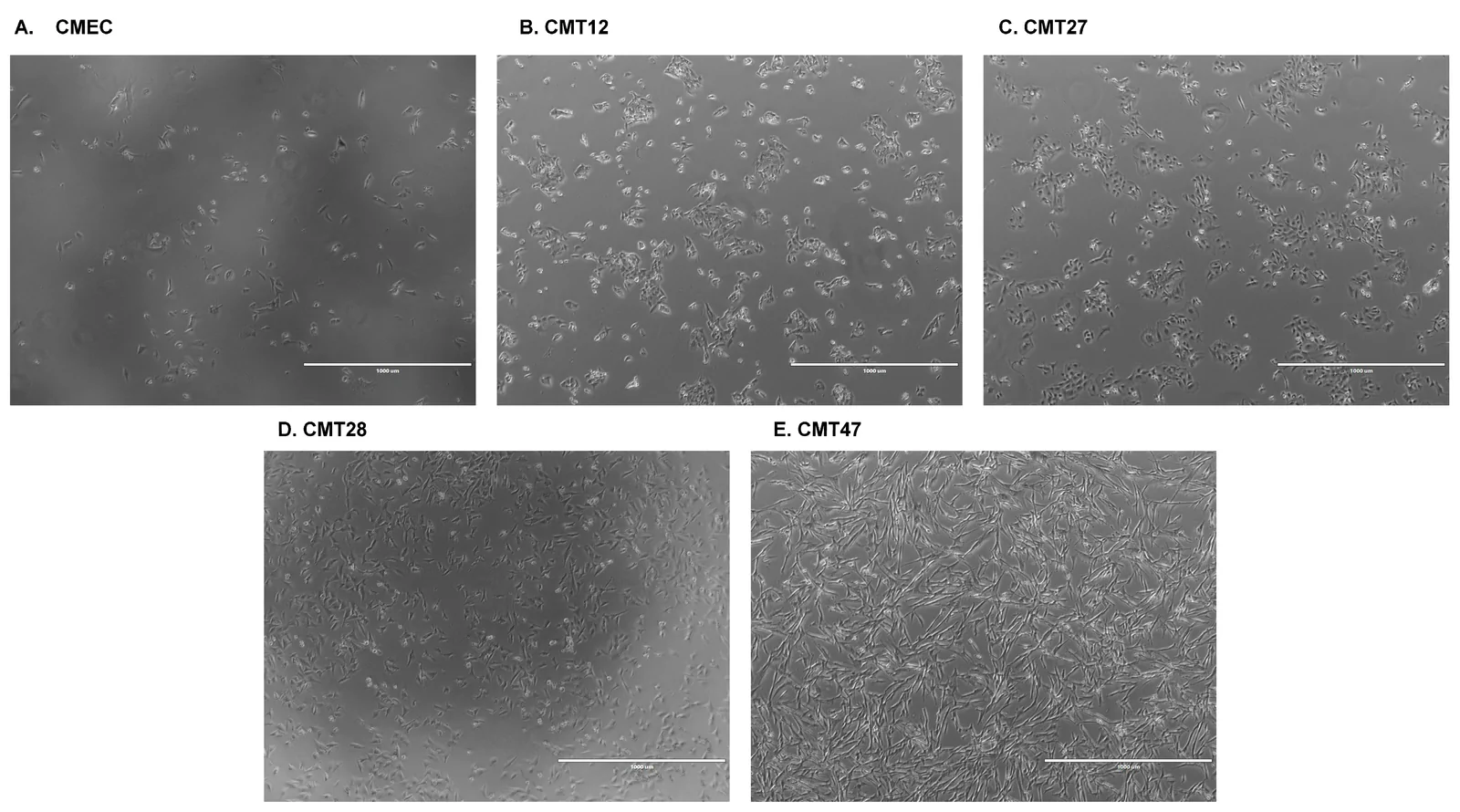
Representative phase-contrast images of canine mammary cell lines used in this study. (**A**) CMEC, (**B**) CMT12, (**C**) CMT27, (**D**) CMT28, and (**E**) CMT47. CMEC represents the normal canine mammary epithelial population, while CMT12, CMT27, CMT28, and CMT47 are tumor-derived cell lines originating from spontaneous canine mammary carcinomas or adenocarcinomas. The cell lines differ in breed of origin and exhibit distinct morphological characteristics consistent with their cellular background. Scale bars = 1000 µm.

This study included four tumor-derived canine mammary cell lines CMT12, CMT27, CMT28, and CMT47 and a normal primary canine mammary epithelial cell population. All cultures showed adherent growth and distinct cellular morphologies. Representative phase-contrast images are shown in Figure 1.

### 3.2. ESR1 Expression Across Canine Mammary Cell Lines

**Figure 2:**
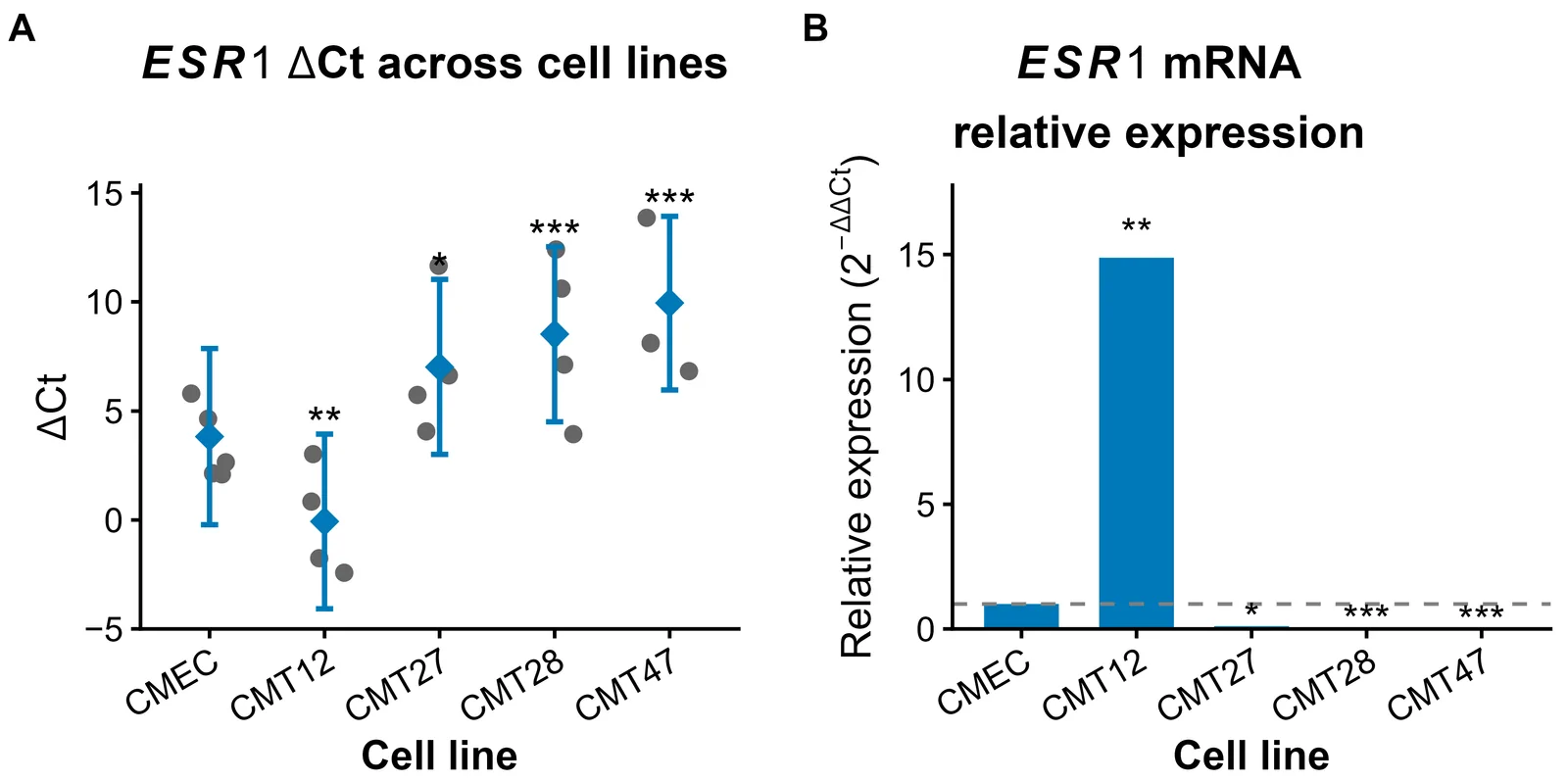
*ESR1* mRNA expression varied widely across canine mammary cell lines. (**A**) ΔCt values for *ESR1* mRNA in CMEC, CMT12, CMT27, CMT28, and CMT47. Each gray point represents the mean ΔCt value from one independent experimental run (*n* = 4). Blue diamonds show the estimated marginal means, and error bars show the 95% confidence intervals. Lower ΔCt values indicate higher *ESR1* expression. (**B**) Model-estimated relative *ESR1* expression calculated using the 2^−ΔΔCt^ method, with CMEC set to 1 (dashed line). Comparisons with CMEC were performed using Dunnett-adjusted contrasts. Significance: * *p* < 0.05; ** *p* < 0.01; *** *p* < 0.001. Model-estimated relative expression values were 1.00 for CMEC, 14.87 for CMT12, 0.109 for CMT27, 0.0387 for CMT28, and 0.0144 for CMT47.

Because *ESR1* mRNA expression can vary among mammary tumors, we first assessed endogenous *ESR1* mRNA expression in the CMT cell lines using CMEC as the normal canine mammary epithelial reference. *ESR1* mRNA expression in the cell lines was assessed by RT-qPCR using *GAPDH* as the reference gene. ΔCt was calculated as Ct (*ESR1*)–Ct (*GAPDH*). The ΔCt values were analyzed using a mixed-effects model with experimental run included as a random effect. *ESR1* mRNA expression was significantly different between CMT cell lines and CMEC *(p* < 0.001; Figure 2A). CMT12 showed lower ΔCt values than CMEC, indicating higher *ESR1* mRNA expression (*p* = 0.0039), whereas CMT27, CMT28, and CMT47 showed higher ΔCt values, indicating lower *ESR1* mRNA expression (*p* = 0.0152, *p* = 0.0009, and *p* = 0.0002, respectively). Fold-change values (relative expression) were calculated using the 2^−ΔΔCt^ method, with CMEC used as the reference. *ESR1* mRNA expression in CMT12 was approximately 14.9 times higher than in CMEC. In CMT27, CMT28, and CMT47, *ESR1* mRNA expression was approximately 9-, 26-, and 70-fold lower, respectively, compared with CMEC (Figure 2B). Overall, *ESR1* mRNA expression was much lower in three of the four tumor-derived cell lines, while CMT12 showed significantly higher *ESR1* expression.

### 3.3. Predicted miR-18a and miR-19a Seed-Match Sites in the Canine ESR1 3′UTR

**Figure 3:**
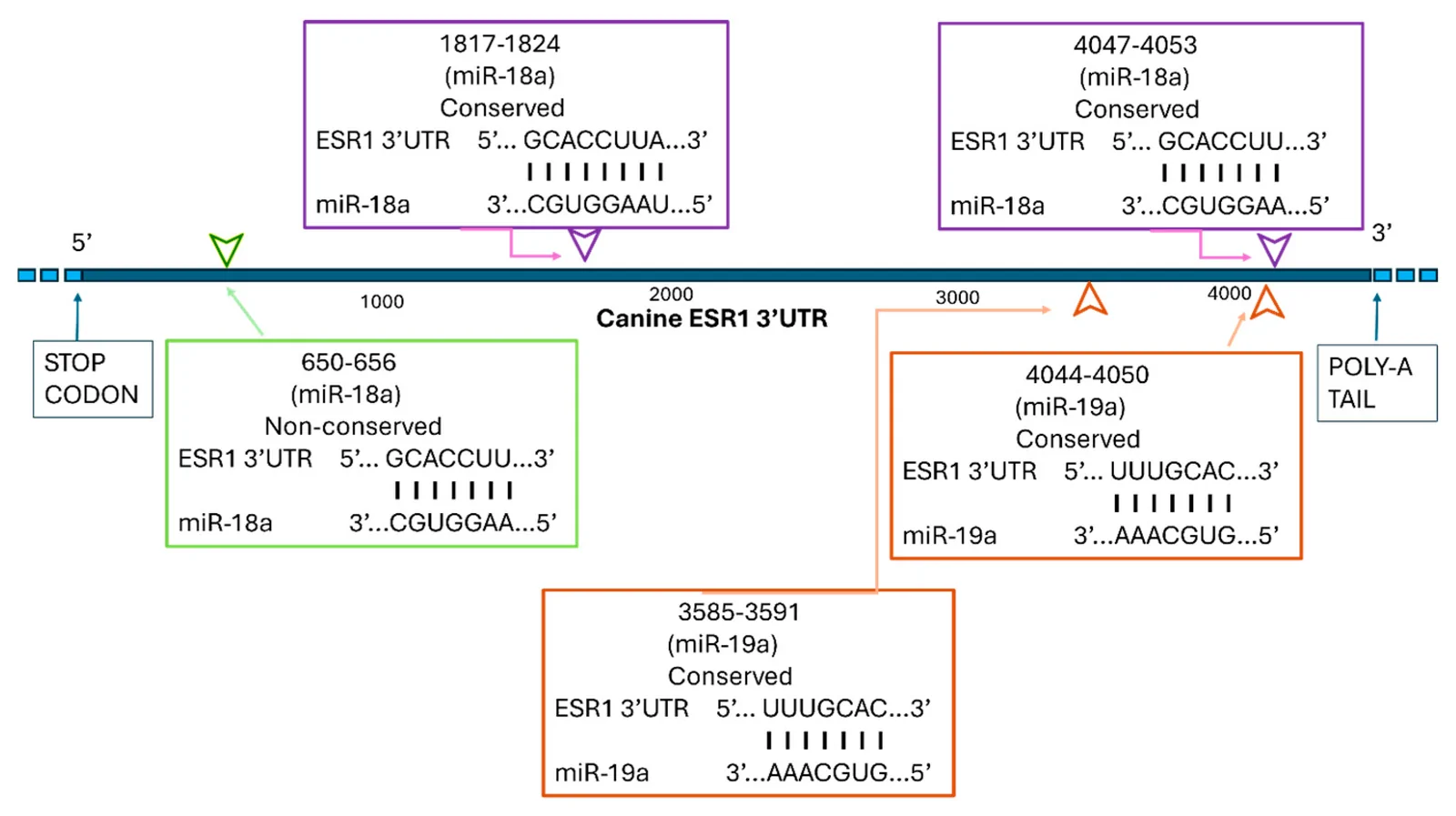
Predicted miR-18a and miR-19a seed-match sites within the canine *ESR1* 3′ untranslated region (3′UTR). Schematic representation of the canine *ESR1* 3′UTR (NM_001286958.2) showing predicted seed-match sites for miR-18a and miR-19a. Conserved miR-18a seed-match sites were identified at nucleotide positions 1817–1824 and 4047–4053, with an additional poorly conserved site at 650–656. Conserved miR-19a seed-match sites were identified at nucleotide positions 3585–3591 and 4044–4050. Enlarged panels show the predicted miRNA–mRNA seed-match alignments. Arrowheads show the predicted seed-match sites. Conserved miR-18a sites are shown in purple, the poorly conserved miR-18a site in green, and conserved miR-19a sites in Orange. Matching arrows link each enlarged alignment to its positions in the 3′UTR. The stop codon marks the transition from the coding sequence (CDS) to the 3′UTR, and the poly(A) tail is shown at the 3′ end of the transcript.

TargetScan analysis identified both conserved and poorly conserved seed-match sites within the canine *ESR1* 3′UTR (NM_001286958.2) (Figure 3). For miR-18a, conserved sites were located at 1817–1824 and 4047–4053, and a poorly conserved site was predicted at 650–656 [33,34]. For miR-19a, conserved sites were located at 3585–3591 and 4044–4050 [33,34]. These predicted binding regions were used to guide the design of the *ESR1* 3′UTR reporter constructs used in the functional assays.

### 3.4. miR-18a Expression Across Canine Mammary Cell Lines

**Figure 4:**
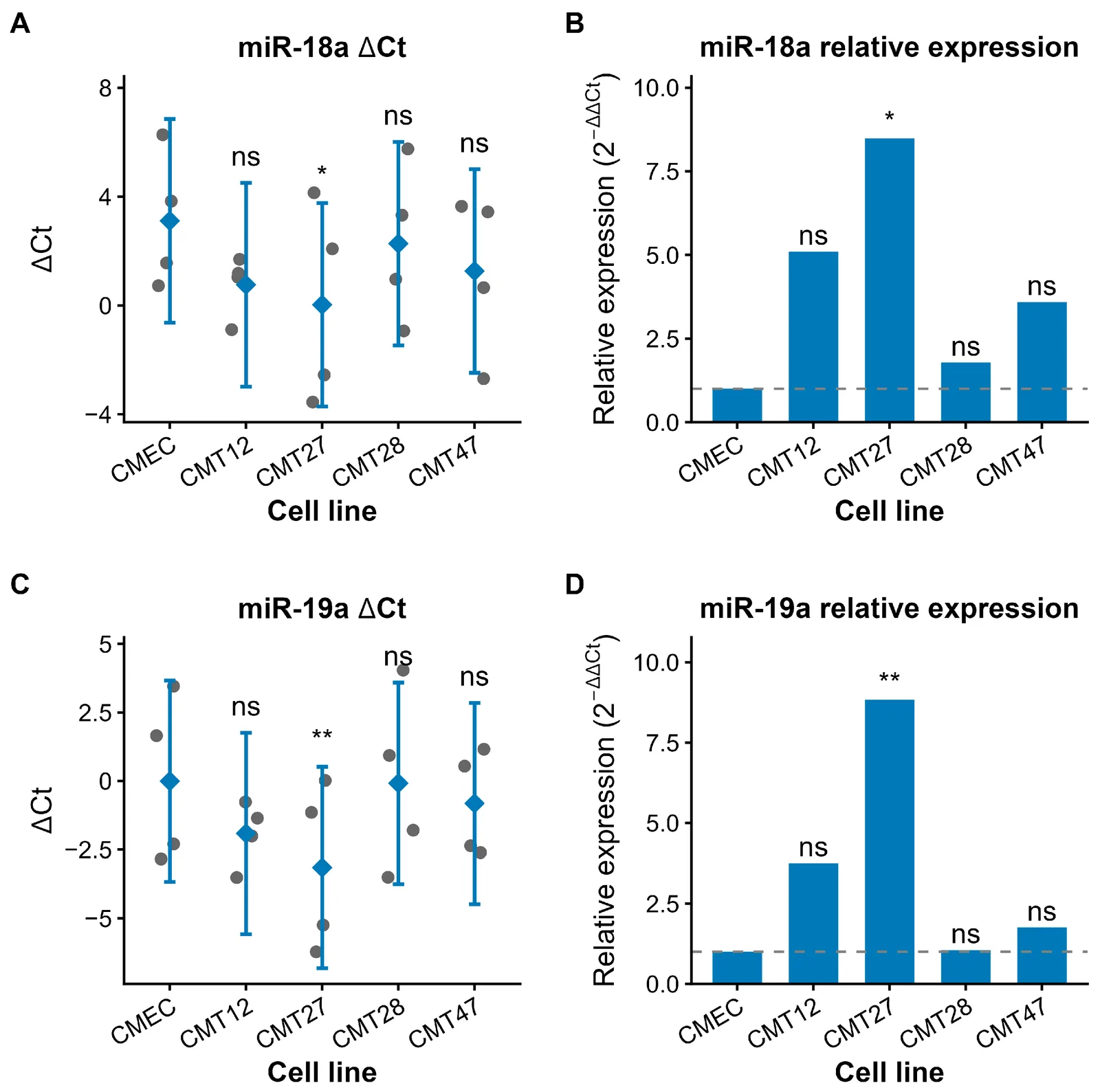
miR-18a and miR-19a expression across canine mammary cell lines. (**A**) ΔCt values for miR-18a and (**B**) model-estimated relative miR-18a expression. (**C**) ΔCt values for miR-19a and (**D**) model-estimated relative miR-19a expression. Each gray point in panels (**A**) and (**C**) represents one independent experimental run (*n* = 4). Blue diamonds represent the estimated marginal mean, and error bars represent 95% confidence intervals. Lower ΔCt values indicate higher miRNA expression. Relative expression in panels (**B**) and (**D**) was calculated using the 2^−ΔΔCt^ method, with CMEC set as the reference value of 1 (dashed line). Technical replicates were averaged within each experimental run before analysis. Comparisons with CMEC were performed using Dunnett-adjusted contrasts. ns, not significant; * *p* < 0.05; ** *p* < 0.01.

Following *ESR1* expression analysis, miR-18a expression was evaluated across the same cell lines using U6 snRNA as the endogenous control. miR-18a expression differed significantly among the cell lines (*F* (4, 12) = 3.68, *p* = 0.0352; Figure 4A). Dunnett comparison showed that miR-18a expression was 8.49-fold higher in CMT27 than in CMEC (adjusted *p* = 0.0172; Figure 4B). CMT12 showed a 5.10-fold increase, but this difference was not significant (adjusted *p* = 0.0735). Expression was also higher in CMT28 and CMT47, but these differences were not significant (adjusted *p* = 0.7372 and *p* = 0.1872, respectively).

Model-estimated fold-change values (2^−ΔΔCt^): miR-18a: CMEC = 1.00; CMT12 = 5.10; CMT27 = 8.49; CMT28 = 1.79; CMT47 = 3.597. miR-19a: CMEC = 1.00; CMT12 = 3.759; CMT27 = 8.84; CMT28 = 1.052; CMT47 = 1.754.

### 3.5. miR-19a Expression Across Canine Mammary Cell Lines

miR-19a expression was then evaluated across the same cell lines using U6 for normalization. miR-19a expression differed significantly among the cell lines (*F* (4, 12) = 6.15, *p* = 0.0063; Figure 4C). Dunnett-adjusted comparisons showed that miR-19a expression was 8.84-fold higher in CMT27 than in CMEC (adjusted *p* = 0.0049; Figure 4D). CMT12, CMT28, and CMT47 did not differ significantly from CMEC (adjusted *p* = 0.0885, *p* = 0.9985, and *p* = 0.6588, respectively).

Overall, the clearest increases in miR-18a and miR-19a expression were observed in CMT27. Based on these expression patterns and the predicted *ESR1* binding sites, we next examined whether these miRNAs could affect reporter activity through selected regions of the *ESR1* 3′UTR.

### 3.6. ESR1 3′UTR Reporter and miRNA Expression Vectors

To examine the effects of miR-18a and miR-19a on the selected regions of the *ESR1* 3′UTR, we used luciferase reporter and miRNA expression plasmid constructs designed for functional analysis. The *ESR1*-WT reporter contained selected fragments of the canine *ESR1* 3′UTR that included the predicted miR-18a and miR-19a seed-match regions identified by bioinformatic analysis. A corresponding *ESR1*-MUT reporter contained deletions within the predicted seed-match regions and was used as a negative control. These *ESR1* 3′UTR fragments were placed downstream of the Gaussia luciferase reporter, allowing changes in reporter activity to be evaluated following miRNA expression.

**Figure 5:**
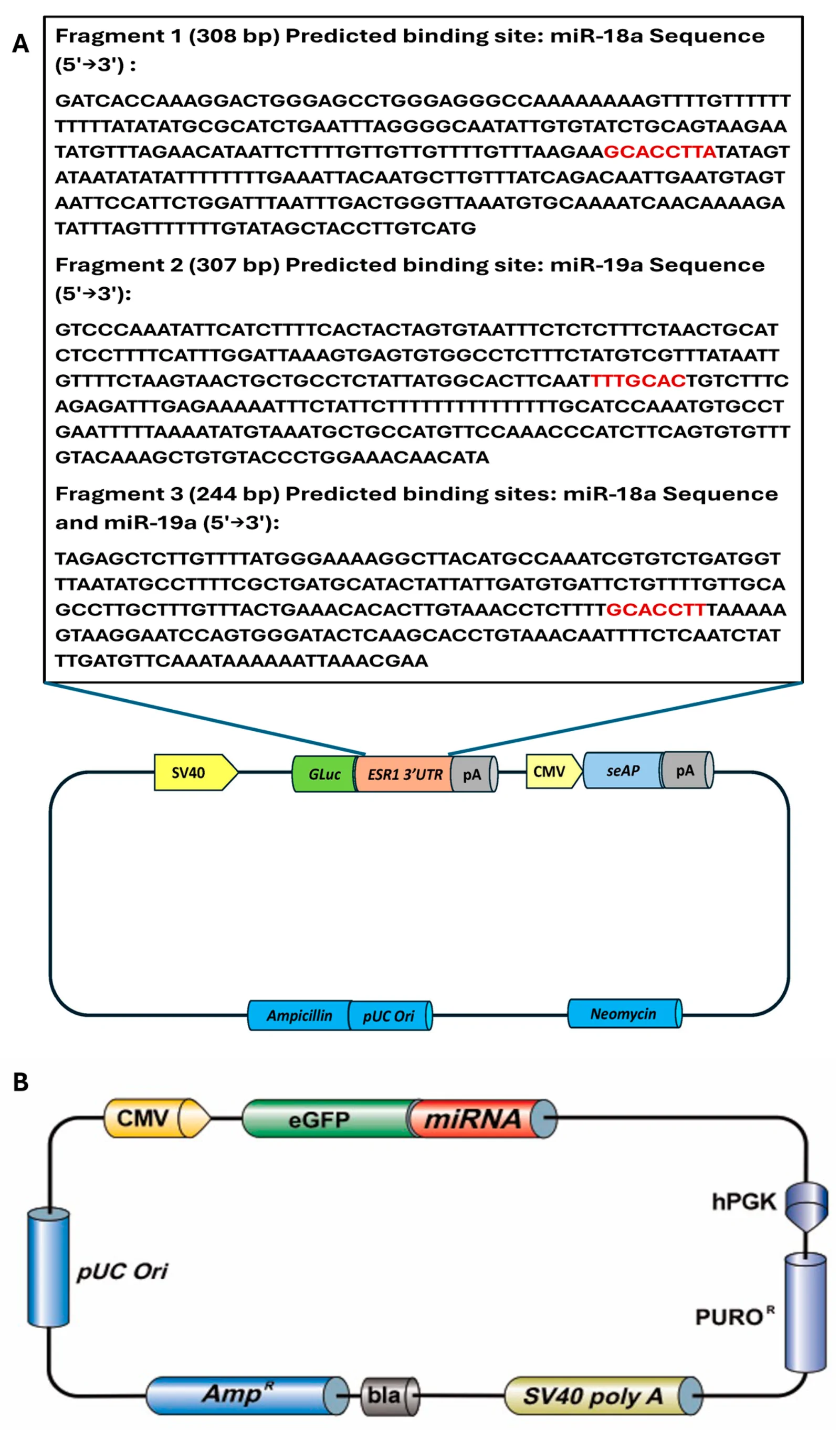
Plasmid constructs used for *ESR1* 3′UTR reporter assays and miRNA expression. (**A**) Schematic of the Gaussia luciferase reporter construct containing three selected regions of the canine *ESR1* 3′UTR with predicted miR-18a and miR-19a seed-match sites. Sequences shown in red indicate the nucleotides deleted from the predicted miRNA seed-match regions in the mutant *ESR1* 3′UTR reporter. The *ESR1* 3′UTR fragments were cloned downstream of the GLuc reporter under the SV40 promoter, with secreted alkaline phosphatase (SEAP) included as an internal control. (**B**) Schematic of the GeneCopoeia miRNA expression vector used for the miR-C control and for expression of miR-18a or miR-19a. The miR-C and miR-18a constructs express eGFP, whereas the miR-19a construct expresses mCherry (not shown in the schematic). Fluorescent reporters were used to confirm reporter expression following transfections. Abbreviations: GLuc, Gaussia luciferase; SEAP, secreted alkaline phosphatase; SV40, simian virus 40 promoter; CMV, cytomegalovirus promoter; pA, polyadenylation signal; pUC Ori, pUC origin of replication; Amp^R^, ampicillin resistance; hPGK, human phosphoglycerate kinase promoter; PURO^R^, puromycin resistance; bla, beta-lactamase gene; miRNA, microRNA; eGFP, enhanced green fluorescent protein; mCherry, red fluorescent protein.

In parallel, GeneCopoeia precursor expression vectors were used to express miR-18a and miR-19a, with fluorescent reporters incorporated into the constructs to visualize vector expression. The miR-C control and miR-18a expression vectors contained an eGFP reporter, whereas the miR-19a expression vector contained a mCherry reporter. Fluorescence imaging was used to confirm expression of the fluorescent reporter proteins following transfection. The overall design of the reporter and miRNA expression constructs is summarized in Figure 5.

### 3.7. Confirmation of Fluorescent Reporter Expression Following miRNA Vector Transfection

**Figure 6:**
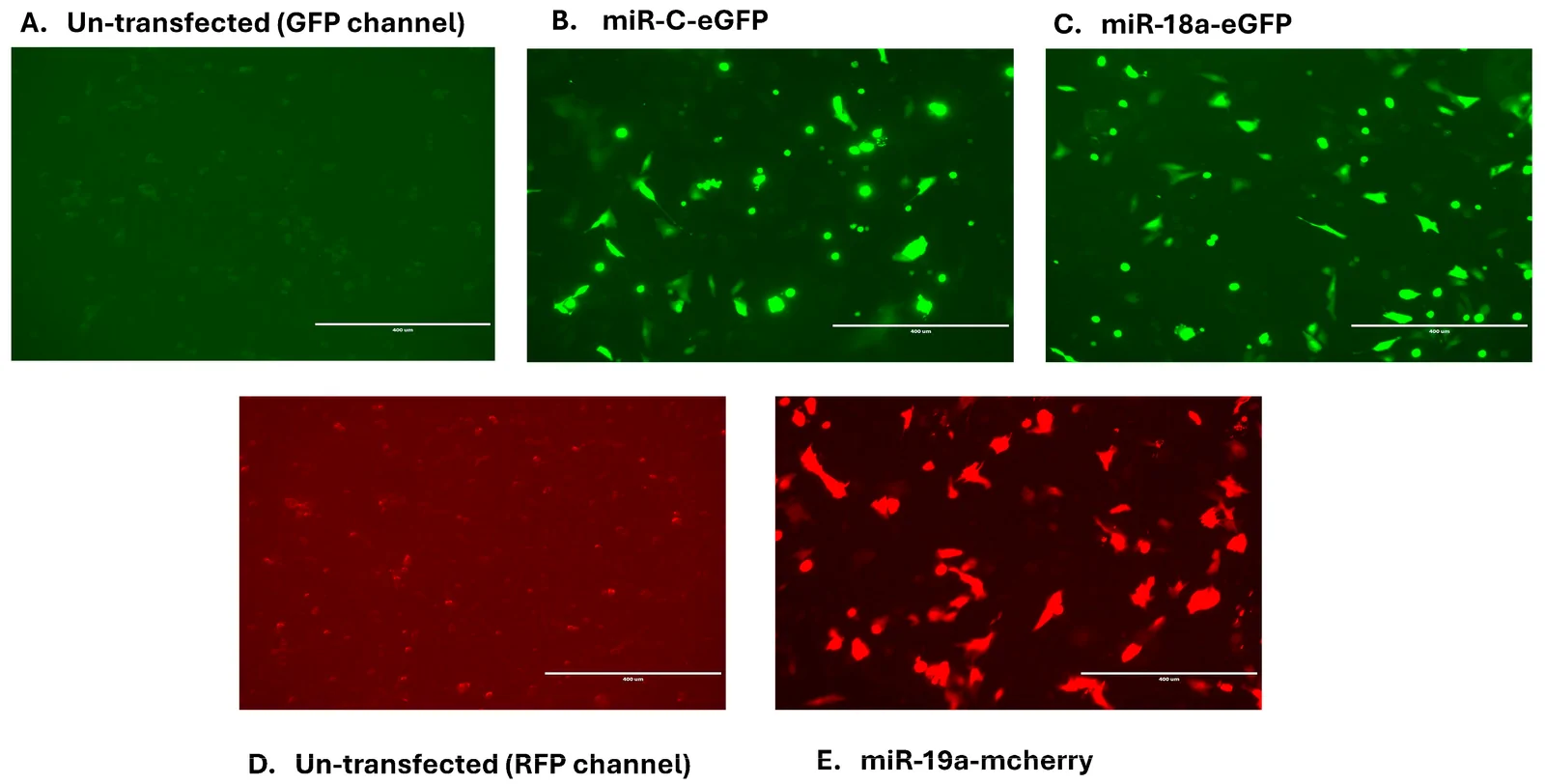
Fluorescence imaging confirms expression of fluorescent reporter proteins from miRNA vectors in CMT28 cells. Panels (**A**) and (**D**) show untransfected CMT28 cells imaged using GFP and RFP channels, respectively, demonstrating background fluorescence. Panel (**B**) shows GFP fluorescence from cells transfected with the miR-C-eGFP vector. miR-C is the GeneCopoeia miRNA control vector used as the negative control for miRNA vector transfection. Panel (**C**) shows GFP fluorescence from cells transfected with the miR-18a-eGFP expression vector. Panel (**E**) shows mCherry fluorescence from cells transfected with the miR-19a-mCherry expression vector. Green shows fluorescence in the GFP channel, and red shows fluorescence in the RFP channel. These images demonstrate reporter expression following transfection of the respective miRNA expression constructs providing indirect evidence of expression from corresponding precursor miRNA vectors from the same promoter. However, mature miR-18a and miR-19a levels were not directly measured. Images were acquired using an EVOS fluorescence imaging system with a 10× objective lens. Scale bars = 400 µm.

Before performing the luciferase reporter assays, fluorescence imaging was used to confirm expression of the fluorescent reporter proteins from the miRNA expression vectors in CMT28 cells. Untransfected cells showed no detectable GFP or mCherry fluorescence, confirming minimal background signal under the imaging conditions. Cells transfected with the miR-C-eGFP control vector showed clear GFP fluorescence. Similarly, cells transfected with miR-18a-eGFP and miR-19a-mCherry showed detectable GFP and mCherry signals, respectively (Figure 6). Because the precursor miRNA and fluorescent reporter proteins were expressed under the same promoter, fluorescence provided indirect evidence of expression from the corresponding precursor miRNA vectors. Levels of mature miR-18a and miR-19a were not directly measured.

### 3.8. miR-18a and miR-19a Reduce ESR1-WT Reporter Activity

**Figure 7:**
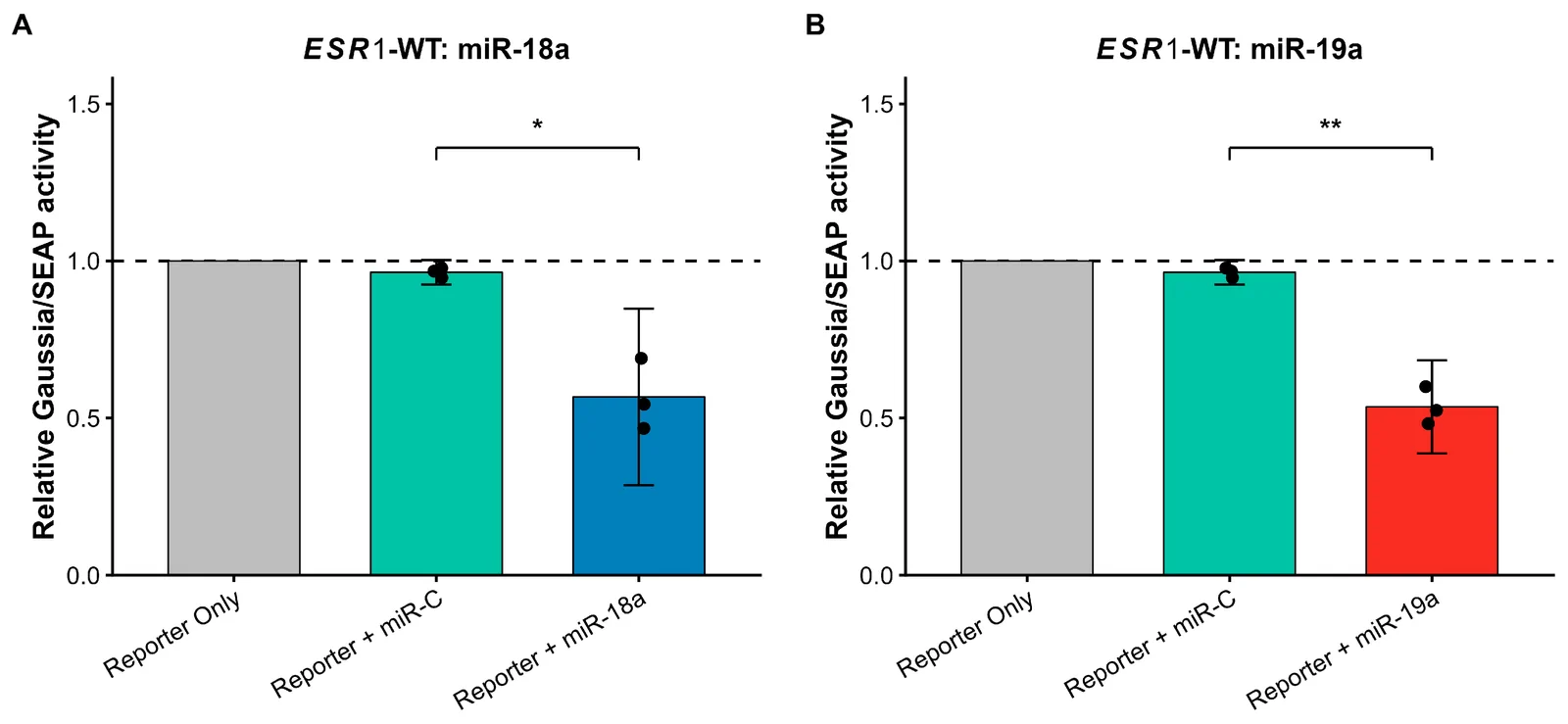
Effect of miR-18a and miR-19a on the *ESR1*-WT reporter in CMT28 cells. CMT28 cells were transfected with the *ESR1*-WT reporter together with miR-C, miR-18a, or miR-19a expression vectors. (**A**) Effect of miR-18a on reporter activity. (**B**) Effect of miR-19a on reporter activity. Gaussia luciferase activity was normalized to SEAP activity. Reporter Only was normalized to 1 (dashed line) and was included as a reference but not in the statistical comparisons. Bars represent the means of three independent experimental runs, error bars represent 95% CIs, and individual points represent the independent runs. Gray bars show Reporter Only, green bars show miR-C, blue bar show miR-18a, and red bar show miR-19a. Technical replicates were averaged within each run. Run-level data and effect estimates with 95% CIs are provided in Supplementary Table S4. Comparisons were performed using two-sided paired *t*-tests. * *p* < 0.05; ** *p* < 0.01.

The effects of miR-18a and miR-19a were tested using the *ESR1*-WT reporter in CMT28 cells. Compared with the miR-C control, miR-18a significantly reduced reporter activity (mean paired difference = −0.397, 95% CI: −0.717 to −0.077; *p* = 0.0334; Figure 7A). miR-19a also significantly reduced reporter activity compared with miR-C (mean paired difference = −0.428, 95% CI: −0.609 to −0.248; *p* = 0.0094; Figure 7B). These results showed that both miR-18a and miR-19a can reduce reporter activity through the selected regions of the canine *ESR1* 3′UTR.

### 3.9. miR-18a and miR-19a Do Not Reduce ESR1-MUT Reporter Activity

**Figure 8:**
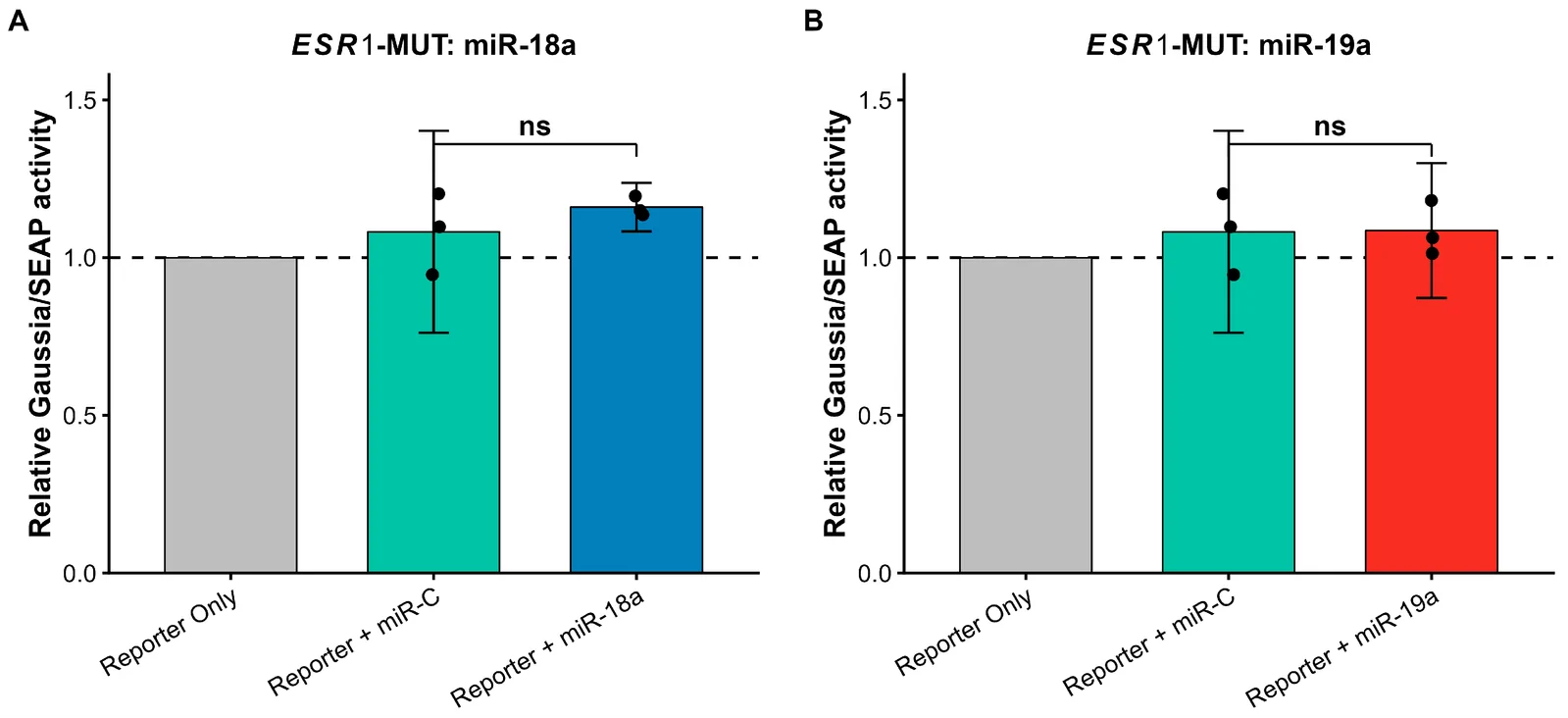
Effect of miR-18a and miR-19a on the *ESR1*-MUT reporter in CMT28 cells. CMT28 cells were transfected with the *ESR1*-MUT reporter together with miR-C, miR-18a, or miR-19a expression vectors. (**A**) Effect of miR-18a on ESR1-MUT reporter activity. (**B**) Effect of miR-19a on ESR1-MUT reporter activity. Gaussia luciferase activity was normalized to SEAP activity. Reporter Only was normalized to 1 (dashed line) and was included as a reference but not in the statistical comparisons. Bars represent the means of three independent experimental runs, error bars represent 95% CIs, and individual points represent the independent runs. Gray bars show Reporter Only, green bars show miR-C, blue bar show miR-18a, and red bar show miR-19a. Technical replicates were averaged within each run. Comparisons were performed using two-sided paired *t*-tests. ns, not significant.

The *ESR1*-Mut reporter was then used to test whether the predicted seed-match regions were important for the miRNA effects. Unlike the *ESR1*-WT, miR-18a did not significantly change *ESR1*-MUT reporter activity compared with miR-C (mean paired difference = 0.078, 95% CI: −0.197 to 0.353; *p* = 0.3467; Figure 8A). Similarly, miR-19a did not significantly change reporter activity (mean paired difference = 0.004, 95% CI: −0.305 to 0.312; *p* = 0.9617; Figure 8B).

The two-way repeated-measures ANOVA showed a significant interaction between reporter construct and miRNA treatment (*F* (2, 4) = 17.89, *p* = 0.0101). The miRNA effects were directly compared between *ESR1*-WT and *ESR1*-MUT reporters. Significant differences were found for both miR-18a (mean difference in effect = −0.475, 95% CI: −0.891 to −0.058; raw *p* = 0.0391; Holm-adjusted *p* = 0.0492) and miR-19a (mean difference in effect = −0.432, 95% CI: −0.729 to −0.135; raw *p* = 0.0246; Holm-adjusted *p* = 0.0492). These results suggest that the reduction in reporter activity depends on the predicted miRNA-responsive regions within the selected regions of *ESR1* 3′UTR.

### 3.10. Effect of Anti-miRs on ESR1-WT Reporter Activity

**Figure 9:**
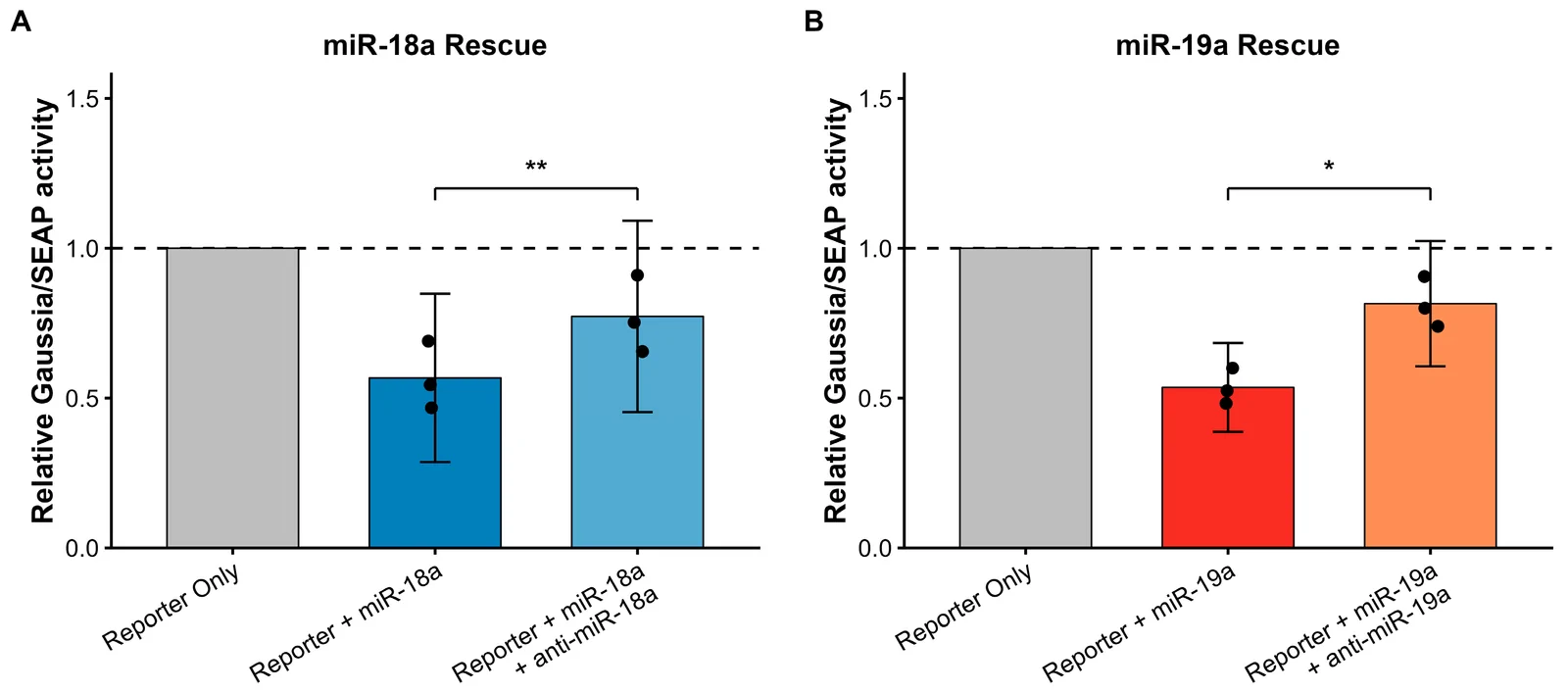
Effect of anti-miR treatment on *ESR1*-WT reporter activity in CMT28 cells. CMT28 cells were transfected with the *ESR1*-WT reporter together with the indicated miRNA, with or without the corresponding anti-miR. (**A**) miR-18a with or without anti-miR-18a. (**B**) miR-19a with or without anti-miR-19a. Gaussia luciferase activity was normalized to SEAP activity. Reporter Only was normalized to 1 (dashed line) and was included as a reference but not in the statistical comparisons. Bars represent the means of three independent experimental runs, error bars represent 95% CIs, and individual points represent the independent runs. Gray bars show Reporter Only. In panel (**A**), blue bar showss miR-18a alone and light blue bar shows miR-18a with anti-miR-18a. In panel (**B**), red bar shows miR-19a alone and orange bar shows miR-19a with anti-miR-19a. Technical replicates were averaged within each run. Each miRNA treatment was compared with the corresponding miRNA plus anti-miR treatment using a two-sided paired *t*-test. * *p* < 0.05; ** *p* < 0.01.

Anti-miRs were then used to test whether blocking each miRNA could increase reporter activity. Addition of anti-miR-18a significantly increased reporter activity compared with miR-18a alone (mean paired difference = 0.205, 95% CI: 0.165 to 0.246; *p* = 0.0021; Figure 9A). Anti-miR-19a also significantly increased reporter activity compared with miR-19a alone (mean paired difference = 0.279, 95% CI: 0.049 to 0.509; *p* = 0.0347; Figure 9B). These results show that anti-miR treatment produced a significant but partial restoration of *ESR1*-WT reporter activity. Together, these findings provide further support for miRNA-dependent regulation of the *ESR1*-WT reporter.

## 4. Discussion

This study examined *ESR1* mRNA expression in canine mammary cell lines and the effects of miR-18a and miR-19a on selected *ESR1* 3′UTR reporter regions. In three of the four tumor-derived cell lines, *ESR1* expression was markedly reduced. miR-18a and miR-19a expression varied among the cell lines, with significant increases observed only in CMT27. The expression patterns did not show a consistent inverse relationship between these miRNAs and *ESR1* across all cell lines. Using expression analysis, target prediction, and functional reporter assays, this study provides reporter-based evidence that miR-18a and miR-19a can repress reporter activity through selected regions of the canine *ESR1* 3′UTR.

Significant variation in *ESR1* expression was also observed across the cell lines used in this study. CMT12 showed higher *ESR1* expression, while CMT27, CMT28, and CMT47 showed much lower expression. This variation is similar to the heterogeneity in estrogen receptor expression reported in human breast cancer [35], which is associated with differences in tumor behavior and treatment response [36]. The low *ESR1* mRNA expression in CMT27, CMT28, and CMT47 suggests that reduced *ESR1* expression may be a common feature in these CMT-derived cell lines, consistent with earlier reports of aggressive, hormone-independent disease in dogs [37,38].

The expression patterns of miR-18a and miR-19a did not show a consistent inverse relationship with *ESR1* expression across all cell lines. Both miRNAs were strongly elevated in CMT27, which also showed low *ESR1* expression. In CMT12, miR-18a and miR-19a expression was numerically higher than in CMEC, but these differences were not statistically significant, while *ESR1* expression remained high. Therefore, miR-18a and miR-19a alone can only explain part of the differences in *ESR1* expression. Additional regulatory mechanisms likely contribute to the control of *ESR1* mRNA expression. A previous study by Abbate et al. reported increased miR-18a expression together with reduced *ESR1* mRNA and ERα immune-expression in highly proliferating canine mammary carcinomas [20]. This is similar to the pattern observed in CMT27 in our study, but lack of a consistent inverse expression pattern in the other cell lines indicates that the relationship between miR-18a and *ESR1* may vary among CMT models and may be more complex.

Computational prediction identified *ESR1* as a potential target of miR-18a and miR-19a. The miRDB database gave high prediction scores for both miRNAs [27]. Because prediction alone cannot confirm regulation, we tested their effects using the canine *ESR1* 3′UTR reporter.

The reporter assays showed that miR-18a and miR-19a reduced activity of the *ESR1*-WT reporter. This is consistent with reports from human cancer studies linking members of the miR-17–92 cluster with reduced ERα expression and *ESR1* signaling [19,39]. In contrast, neither miRNA significantly reduced activity of the mutant reporter containing deletions in the predicted seed-match regions. The effects of both miRNAs were also significantly different between the *ESR1*-WT and *ESR1*-MUT reporters. Blocking each miRNA with the corresponding anti-miR partially increased reporter activity compared with the miRNA alone. These findings indicate that the observed repression depends on the selected miRNA-responsive regions of the canine *ESR1* 3′UTR. To our knowledge, this is the first functional reporter evidence that miR-18a and miR-19a can reduce reporter activity through selected regions of the canine *ESR1* 3′UTR in CMT cells.

Reduced ERα expression is commonly seen in more aggressive mammary tumors in both humans and dogs, and ER-negative tumors tend to grow more rapidly and respond poorly to hormone-based therapies [8,40]. Our reporter findings suggest that miR-18a and miR-19a have the potential to contribute to *ESR1* regulation in CMTs, consistent with experimental evidence of *ESR1* regulation by these miRNAs in human systems [39]. However, this requires validation at the endogenous level. These miRNAs have also been investigated as possible biomarkers for tumor classification or prognosis [41], although this would require validation in larger groups of dogs.

Understanding *ESR1* regulation in CMTs could be useful for future studies of diagnosis and treatment. If miRNA-mediated repression contributes to reduced *ESR1* expression, miRNA profiles may help distinguish different tumor patterns or subtypes. However, the present study does not establish any diagnostic or therapeutic use of miR-18a and miR-19a. These possible applications require further validation.

A limitation of this study is the small number of canine mammary cell lines examined and use of only CMT28 for the functional reporter experiments. In addition, the CMEC reference cells were derived from a single dog and may not represent the biological variation among normal canine mammary tissues. Although these cell lines showed clear differences in *ESR1* and miRNA expression, studies using additional cell lines, primary CMT samples, and in vivo models will be needed to determine whether the same patterns occur more broadly. In addition, the luciferase reporter experiments were designed specifically to test the selected miR-18a and miR-19a responsive regions of the *ESR1* 3′UTR. The reporter assays therefore demonstrate an effect on the reporter construct but do not establish whether these miRNAs regulate endogenous *ESR1* mRNA or ERα protein in intact tumor cells. Fluorescence provided indirect evidence of the precursor miRNA vector expression. Mature miR-18a, miR-19a levels and inhibitors efficiency were not directly assessed in the reporter experiments. A non-targeting anti-miR control was not included; therefore, non-specific effects of the additional oligonucleotide or transfection procedure cannot be excluded. The reporter assays included only three independent experimental runs, which limits the precision of the estimates. Therefore, the results should be interpreted with caution. Other miRNAs, RNA-binding proteins, or epigenetic mechanisms may also contribute to the differences in *ESR1* expression observed among the cell lines.

Overall, miR-18a and miR-19a repressed reporter activity through selected regions of the canine *ESR1* 3′UTR. The response to miRNA treatment differed significantly between the *ESR1*-WT and *ESR1*-MUT reporters. Anti-miR treatment also partially increased *ESR1*-WT reporter activity. These findings support the involvement of the selected miRNA-responsive regions. The importance of this regulation in endogenous *ESR1* expression remains to be determined. Future studies should examine whether inhibition of endogenous levels of miR-18a and miR-19a can increase or restore endogenous levels of *ESR1* mRNA. The effects of miR inhibition on cellular behavior should also be investigated.

## 5. Conclusions

This study demonstrates that miR-18a and miR-19a can reduce the activity of a reporter containing selected regions of the canine *ESR1* 3′UTR. The response to miRNA treatment differed significantly between the *ESR1*-WT and *ESR1*-MUT reporters. Addition of the corresponding anti-miRs were able to partially re-store the activity of the *ESR1*-WT reporter in the presence of miR-18a or miR-19a. Further functional experiments will be needed to demonstrate that the same regulation helps manage endogenous *ESR1* mRNA or ERα protein levels in additional cell lines and primary tumor cells or in vivo models. Such further validation will be required to establish these miRNAs as significant biomarkers or targetable molecules.

## Figures and Tables

**Table 1:** Primer sequences and amplicon sizes used for canine mRNA RT-qPCR.

Canine Gene	Forward Primer (5′ → 3′)	Reverse Primer (5′ → 3′)	Amplicon Size (bp)
*GAPDH*	ATGGTGAAGGTCGGAGTCAA	ATCACCCCATTTGATGTTGG	261
*ESR1*	ATGTGTCCAGCTACCAACC	GTCTTTCCGTATCCCGCC	117

## Data Availability

The original contributions presented in this study are included in the article/Supplementary Materials. Further inquiries can be directed to the corresponding author.

## Supplementary Materials

The following supporting information can be downloaded at: https://www.mdpi.com/article/10.3390/vetsci13101071/s1, Figure S1: RT-qPCR standard curves for (A) *GAPDH* and (B) *ESR1*; Figure S2: RT-qPCR standard curves for (A) miR-18a, (B) miR-19a, and (C) U6; Table S1: *ESR1* 3′UTR luciferase reporter plasmids and precursor miRNA expression vectors; Table S2: Anti-miR oligonucleotides used in this study; Table S3: Selected, non-contiguous *ESR1* 3′UTR fragments used in the luciferase reporter assays; Table S4: Run-level luciferase reporter data and effect estimates with 95% confidence intervals.
