# Supplementary material for: MicroRNA Regulation of the Estrogen Receptor Alpha 3′UTR in Canine Mammary Tumor (CMT28) Cells
Source: Vet Sci. 2026 Oct 10;13(10):1071. doi: 10.3390/vetsci13101071 (PMC13709629; doi:10.3390/vetsci13101071)
Supplement: Supplementary file 1 [file vetsci-13-01071-s001.zip › vetsci-4571995-supplementary.pdf]

Supplementary Materials

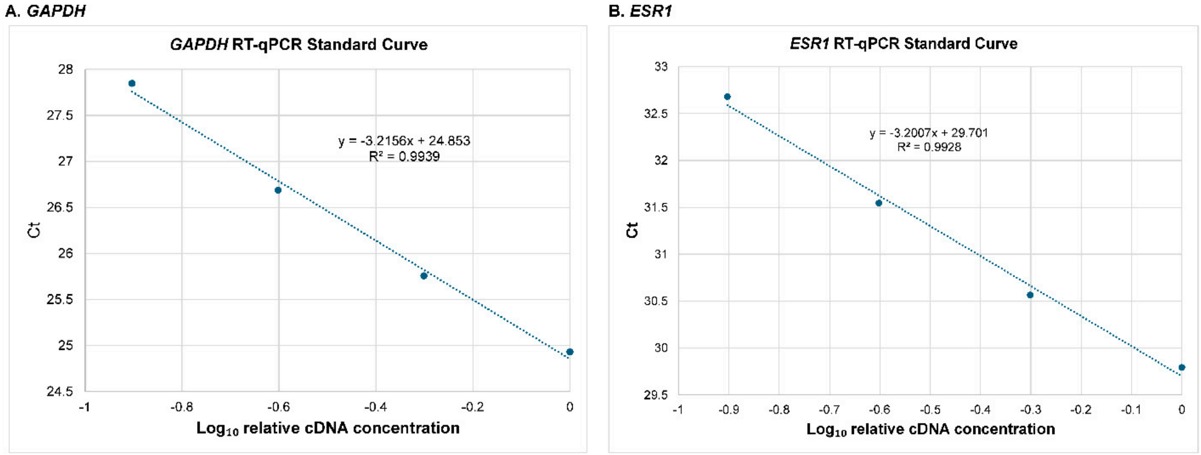

Supplementary Figure S1. RT-qPCR standard curves for (A) *GAPDH* and (B) *ESR1*. The slopes were −3.2156 and −3.2007, the R^2 values were 0.9939 and 0.9928, and the calculated amplification efficiencies were 104.6% and 105.3%, respectively.

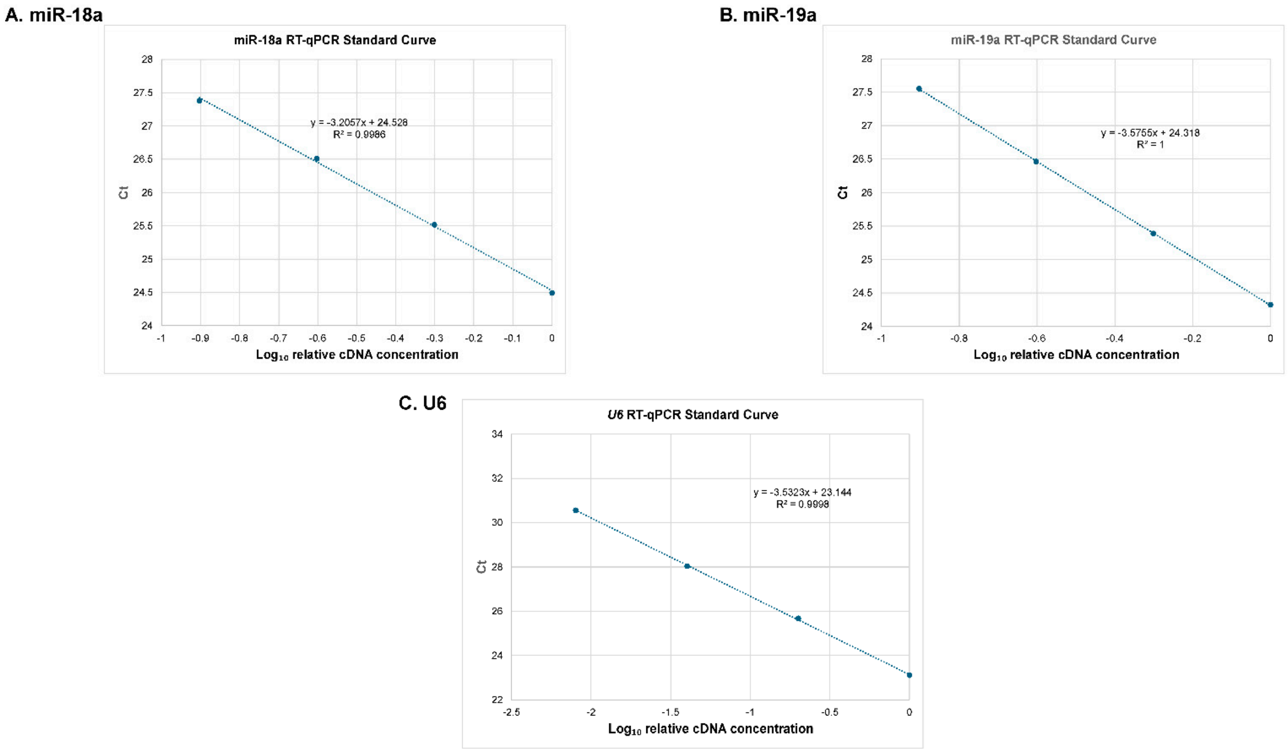

Supplementary Figure S2. RT-qPCR standard curves for (A) miR-18a, (B) miR-19a, and (C) U6. The slopes were −3.2057, −3.5755, and −3.5323; the R^2 values were 0.9986, 1.0000, and 0.9998; and the calculated amplification efficiencies were 105.1%, 90.4%, and 91.9%, respectively.

Supplementary Table S1. *ESR1* 3'UTR luciferase reporter plasmids and precursor miRNA expression vectors.

**Plasmid**	**Catalog No.**	**Description**	**Vector Backbone**	**Insert Length (bp)**	**Plasmid Size (bp)**
Wild-type *ESR1* 3'UTR reporter Containing selected non-contiguous regions. (ESR1-WT)	CS-miT740T-MT05-01	Three selected noncontiguous regions of the Wild-type *Canis lupus familiaris ESR1* 3'UTR (NM_001286958.2) containing predicted miR-18a and miR-19a seed-match sites cloned	pEZX-MT05	859	9422

		downstream of Gaussia luciferase			
Mutant *ESR1* 3'UTR reporter (ESR1-Mut)	CS-miT740T-MT05-02	Corresponding selected-region reporter containing deletions at positions 151–158, 459–465, and 766–772 of the reporter insert.	pEZX-MT05	837	9400
miR-C control vector	CmiR0001-MR04	Negative control miRNA expression vector (eGFP reporter)	pEZX-MR04	—	5018
miR-18a precursor vector	CS-miR738T-MR04-01	Precursor cfa-miR-18a clone with eGFP and puromycin	pEZX-MR04	92	4898
miR-19a precursor vector	CS-miR739T-MR04-01	Precursor cfa-miR-19a clone with mCherry and hygromycin	pEZX-MR04	58	5280

Supplementary Table S2. Anti-miR oligonucleotides used in this study.

Reagent	Catalog No.	Sequence (5'→3')	Modification	Amount	Purification	Target miRNA
Anti-miR-18a	HmiR-AN0255-SN-5	CUAUCUGCACUA GAUGCACCUUA	2'-O-methyl RNA	5 nmol	HPLC	hsa-miR-18a-5p
Anti-miR-19a	HmiR-AN0293-SN-5	UCAGUUUUGCAU AGAUUUGCACA	2'-O-methyl RNA	5 nmol	HPLC	hsa-miR-19a-3p

Note: Although the inhibitors were designed for human miR-18a-5p and miR-19a-3p, the mature sequences of both miRNAs are identical in humans and dogs. Therefore, the inhibitors are also complementary to cfa-miR-18a-5p (MIMAT0009832) and cfa-miR-19a-3p (MIMAT0006650).

Supplementary Table S3. Selected, non-contiguous *ESR1* 3'UTR fragments used in the luciferase reporter assays.

S3A. Positions of predicted miRNA seed-match sites in the native *ESR1* 3'UTR and corresponding deletion positions used to generate the *ESR1*-MUT reporter.

Fragment	Predicted site	Position in native *ESR1* 3′UTR	Deletion position in reporter insert

Fragment 1	miR-18a	1817–1824	151–158
Fragment 2	miR-19a	3585–3591	459–465
Fragment 3	miR-19a	4044–4050	766–772
Fragment 3	miR-18a	4047–4053	766–772

The predicted miR-18a and miR-19a seed-match sites in Fragment 3 overlap. Therefore, the deletion at positions 766–772 of the reporter insert disrupted both sites.

S3B: S3B. Sequences of the selected *ESR1* 3'UTR fragments included in the reporter. The *ESR1*-WT reporter contained three selected, non-overlapping fragments with a combined length of 859 bp. These fragments did not represent the complete native canine *ESR1* 3'UTR. **The sequences deleted to generate the *ESR1*-MUT reporter are shown in red.**

Fragment 1 (308 bp)
Predicted seed-match region(s): miR-18a
Sequence (5'→3'):

GATCACCAAAGGACTGGGAGCCTGGGAGGGCCAAAAAAAAGTTTTGTTTTTTTTTTTATATATGCG
CATCTGAATTTAGGGGCAATATTGTGTATCTGCAGTAAGAATATGTTTAGAACATAATTCTTTTGT
TGTTGTTTTGTTTAAGAAGCACCTTATATAGTATAATATATATTTTTTTTGAAATTACAATGCTTG
TTTATCAGACAATTGAATGTAGTAATTCCATTCTGGATTTAATTTGACTGGGTTAAATGTGCAAAA
TCAACAAAAGATATTTAGTTTTTTTGTATAGCTACCTTGTCATG

Fragment 2 (307 bp)
Predicted seed-match region(s): miR-19a
Sequence (5'→3'):

GTCCCAAATATTCATCTTTTCACTACTAGTGTAATTTCTCTCTTTCTAACTGCATCTCCTTTTCAT
TTGGATTAAAGTGAGTGTGGCCTCTTTCTATGTCGTTTATAATTGTTTTCTAAGTAACTGCTGCCT
CTATTATGGCACTTCAATTTTGCACTGTCTTTCAGAGATTTGAGAAAAATTTCTATTCTTTTTTTT
TTTTTTTGCATCCAAATGTGCCTGAATTTTTAAAATATGTAAATGCTGCCATGTTCCAAACCCATC
TTCAGTGTGTTTGTACAAAGCTGTGTACCCTGGAAACAACATA

Fragment 3 (244 bp)
Predicted seed-match region(s): miR-18a and miR-19a
Sequence (5'→3'):

TAGAGCTCTTGTTTTATGGGAAAAGGCTTACATGCCAAATCGTGTCTGATGGTTTAATATGCCTTTT
CGCTGATGCATACTATTATTGATGTGATTCTGTTTTGTTGCAGCCTTGCTTTGTTTACTGAAACACA
CTTGTAAACCTCTTTTGCACCTTTAAAAAGTAAGGAATCCAGTGGGATACTCAAGCACCTGTAAACA
ATTTTCTCAATCTATTTGATGTTCAAATAAAAAATTAAACGAA

Supplementary Table S4. Run-level luciferase reporter data and effect estimates with 95% confidence intervals.

Table S4A: Run-level data

Run	UTR	miR	Relative Activity
1	ESR1-MUT	Reporter Only	1
2	ESR1-MUT	Reporter Only	1
3	ESR1-MUT	Reporter Only	1
1	ESR1-MUT	miR-18a	1.195241
2	ESR1-MUT	miR-18a	1.149454
3	ESR1-MUT	miR-18a	1.13616
1	ESR1-MUT	miR-19a	1.063476
2	ESR1-MUT	miR-19a	1.013518
3	ESR1-MUT	miR-19a	1.181299
1	ESR1-MUT	miR-C	1.202641
2	ESR1-MUT	miR-C	0.946164
3	ESR1-MUT	miR-C	1.097818
1	ESR1-WT	Reporter Only	1
2	ESR1-WT	Reporter Only	1
3	ESR1-WT	Reporter Only	1
1	ESR1-WT	miR-18a	0.467328
2	ESR1-WT	miR-18a	0.54419
3	ESR1-WT	miR-18a	0.69
1	ESR1-WT	miR-18a + Anti-miR-18a	0.65523
2	ESR1-WT	miR-18a + Anti-miR-18a	0.75272
3	ESR1-WT	miR-18a + Anti-miR-18a	0.91
1	ESR1-WT	miR-19a	0.524601
2	ESR1-WT	miR-19a	0.48221
3	ESR1-WT	miR-19a	0.6
1	ESR1-WT	miR-19a + Anti-miR-19a	0.905685
2	ESR1-WT	miR-19a + Anti-miR-19a	0.73935
3	ESR1-WT	miR-19a + Anti-miR-19a	0.8
1	ESR1-WT	miR-C	0.977353

2	ESR1-WT	miR-C	0.967586
3	ESR1-WT	miR-C	0.946548

Table S4B: Statistical comparisons

Comparison	Mean_Difference	CI_Lower	CI_Upper	P_value	Holm_Adjusted_P
ESR1-WT: miR-18a vs miR-C	-0.39666	-0.7167	-0.07661	0.033414	NA
ESR1-WT: miR-19a vs miR-C	-0.42823	-0.60855	-0.2479	0.009443	NA
ESR1-MUT: miR-18a vs miR-C	0.078077	-0.19722	0.353377	0.346718	NA
ESR1-MUT: miR-19a vs miR-C	0.00389	-0.30452	0.312298	0.961655	NA
miR-18a: ESR1-WT vs ESR1-MUT effect	-0.47473	-0.89119	-0.05828	0.039145	0.049182
miR-19a: ESR1-WT vs ESR1-MUT effect	-0.43212	-0.72918	-0.13505	0.024591	0.049182
miR-18a + anti-miR vs miR-18a	0.205477	0.165072	0.245882	0.002082	NA
miR-19a + anti-miR vs miR-19a	0.279408	0.049445	0.509371	0.034697	NA
